# Academy of Natural Sciences

**Published:** 1841-11-13

**Authors:** 


### PROCEEDINGS OF SOCIETIES.

### ACADEMY OF NATURAL SCIENCES.

Though unusually rich in papers of inportance in technical Natural History, the Bulletin of the last month presents little that is of immediate interest to the Medical Profession. We extract a single article for the information of comparative anatomists. The communication was made at the session of the 12th of October.

Dr. Goddard stated that he had examined the so called "Missourium Kochii," and found it to be a skeleton composed of Mastodon bones, most of which appeared to belong to a single set, many, however, having been superadded, and others mended and glued together in a manner wholly erroneous.

The following errors were especially noticed:

*Spine.*—The spine presented the anomaly of 8 cervical vertebræ; and instead of 19 dorsal and 4 lumbar, had 23 dorsal and 10 lumbar vertebræ, making the number of bones in the spine too great by 11. The bones articulated with the 2d and 4th ribs were cervical vertebræ. The spaces between the vertebræ were much magnified by thick wooden blocks placed between them, and the spine was curved upwards, so as to give an exaggerated idea of the height of the animal.

*Ribs.*—These were redundant in number, and were spread out as much as possible, so as to present the appearance of a wide and flat chest. The first pair of ribs were stuck on the bones of the shoulder, to resemble clavicles—bones which the Mastodon does not possess.

*Head.*—The head was that of a Mastodon with the top deficient, and a piece of an ethmoidal? bone glued on in front to resemble a snout. The tusks were distorted laterally, so as to occupy a space of 18 feet in width.

*Scapulæ and ilia.*—These having been deficient, were very ingeniously pieced out with wood, glued over so as to resemble bone.

*Feet.*—The feet were ludicrously made up of carpal and tarsal bones, and presented the wonderful anomaly of four phalanges to each toe.

Several other discrepancies were observed; apart from which Dr. G. considered the skeleton one of very great interest.

